# Establishing a valid construct of fear of childbirth: findings from in-depth interviews with women and midwives

**DOI:** 10.1186/s12884-019-2241-7

**Published:** 2019-03-18

**Authors:** P. Slade, K. Balling, K. Sheen, G. Houghton

**Affiliations:** 1 Institute of Health and Life Sciences, University of Liverpool, Ground Floor Whelan Building, Brownlow Hill, Liverpool, L69 3GB UK; 2 Natural Sciences and Psychology, Liverpool John Moores University, Tom Reilly Building, Byrom Street, Liverpool, L3 3AF UK; 3 Liverpool Women’s Hospital Foundation Trust, Crown Street, Liverpool, L8 7SS UK

**Keywords:** Antenatal anxiety, Childbirth anxiety, Fear of childbirth, Qualitative tokophobia

## Abstract

**Background:**

Fear of childbirth (FOC) can have a negative impact on a woman’s psychological wellbeing during pregnancy and her experience of birth. It has also been associated with adverse obstetric outcomes and postpartum mental health difficulties. However the FOC construct is itself poorly defined. This study aimed to systematically identify the key elements of FOC as reported by women themselves.

**Methods:**

Semi-structured interviews with pregnant women (*n* = 10) who reported to be fearful of childbirth and telephone interviews with consultant midwives (*n* = 13) who regularly work with women who are fearful of childbirth were conducted. Interviews were analysed using thematic analysis for each group independently to provide two sources of information. Findings were reviewed in conjunction with a third source, a recently published meta-synthesis of existing literature of women’s own accounts of FOC. The key elements of FOC were determined via presence in two out of the three sources at least one of which was from women themselves, i.e. the reports of the women interviewed or the meta-synthesis.

**Results:**

Seven themes were identified by the women and the consultant midwives: *Fear of not knowing and not being able to plan for the unpredictable, Fear of harm or stress to the baby, Fear of inability to cope with the pain, Fear of harm to self in labour and postnatally, Fear of being ‘done to’, Fear of not having a voice in decision making and Fear of being abandoned and alone*. One further theme was generated by the women and supported by the reports included the meta-synthesis: *Fear about my body’s ability to give birth*. Two further themes were generated by the consultant midwives and were present also in the meta-synthesis: *Fear of internal loss of control and Terrified of birth and not knowing why.*

**Conclusions:**

Ten key elements in women’s FOC were identified. These can now be used to inform development of measurement tools with verified content validity to identify women experiencing FOC, to support timely access to support during pregnancy.

**Electronic supplementary material:**

The online version of this article (10.1186/s12884-019-2241-7) contains supplementary material, which is available to authorized users.

## Background

Expectations formed about childbirth before or during pregnancy are key determinants for women’s experience of and behaviour before and during childbirth [1]. Some women experience high levels of fear specific to giving birth, and at its most severe fear of childbirth has been likened to a phobic response (‘tokophobia’) [1, 2].

Although some concerns relating to how a forthcoming birth may be experienced can be considered normal for many women, and may in some instances be potentially adaptive, severe fears of childbirth (or tokophobia) involving extreme fear, worry or concern specific to giving birth [1] is likely to be more problematic. To date, an absence of a clear definition for fear of childbirth and identification of levels that may constitute a phobic response has led to significant heterogeneity in estimations of prevalence [2].

Fear of childbirth holds implications for women’s experiences of pregnancy and birth [3, 4]. Elevated fear during pregnancy has been associated with the progression of birth (longer birth duration), an increased likelihood of intervention including augmentation of labour and emergency caesarean section [5–8] and an increased likelihood of elective caesarean section [9–12]. However studies exploring relationships between fear of childbirth and adverse birth outcomes are inconsistent [7, 13] and further examination is required.

Elevated anxiety and fear during pregnancy holds important implications for both maternal postpartum wellbeing and child development [14, 15]. Although there are parallels between generalised anxiety and fear of childbirth, studies have shown that the two are not synonymous and can be considered separate constructs [16]. Furthermore, assessment of specific fear of childbirth has been identified as a superior predictor of both maternal and infant outcome over generalised anxiety alone [16].

Despite international recognition about the impact and implications of FOC, the construct is poorly defined and current methods of identifying fear during pregnancy are varied [2, 17, 18]. In the United Kingdom, present recommendations include identifying depression and general anxiety during pregnancy [19]. There is currently no routine pathway to ask specifically about women’s fears of childbirth in the United Kingdom and provision of support varies [20, 21]. Furthermore, the dominance of tools developed in Scandinavian populations [17] has led to frequent use of measurement tools that hold uncertain utility for other populations where the focus of fear may vary [22, 23] or where translation into English renders the interpretation of items ambiguous [23, 24].

Reliable and valid identification of high levels of fear early in pregnancy could enable interventions to support and manage these concerns to reduce distress and fear of childbirth. However, in order to ask more specifically about FOC, it is important that a clear, comprehensive and culturally appropriate definition for fear of childbirth is developed. In addition to this, optimal timing of assessment for FOC needs to be informed by the views of women and midwives.

### Aim

To systematically identify the key elements that constitute the fear of childbirth construct. In addition, to identify women’s and midwives’ perspectives on optimal or preferred timing for asking about fears during pregnancy.

## Methods

### Design

The study used a qualitative research design with semi-structured interviews. The interviews were conducted in two stages.

- Stage one consisted of semi-structured interviews with women who reported to be fearful of childbirth.
- Stage two consisted of semi-structured telephone interviews with consultant midwives who regularly support women who are fearful of childbirth.

### Setting

Interviews with women were either conducted at the Liverpool Women’s Hospital NHS Foundation Trust (LWHFT) (*n* = 1), or in the woman’s home (*n* = 9), the choice of venue was guided by the women’s preference to ensure maximum comfort for the woman. All consultant midwife interviews were conducted by telephone (*n* = 13).

### Ethical approval

Ethical approval was sought and obtained from the University of Liverpool (15/NW/0922) and the study was sponsored by University of Liverpool (UoL00177).

### Participants

#### Stage one

Pregnant women under the care of the consultant midwife for reasons relating to fear of childbirth, who were aged over 16 years and fluent in spoken English were eligible to participate. Women who had a history of stillbirth or intrauterine death, an ongoing serious maternal medical condition, where there was a medical concern for the baby in their current pregnancy, or if they were under the care of the fetal medicine unit, the perinatal mental health team or the enhanced midwifery team were not eligible to participate.

The timing of recruitment was pragmatic; women who reported to be experiencing a fear of childbirth as part of standard care were referred by their midwives to the consultant midwife at LWHFT. Their fear of childbirth was then assessed by the consultant midwife during a routine clinical appointment. The research interviews were arranged, at the woman’s earliest convenience, after the consultant midwife had gained consent. All women were interviewed in the 3rd trimester. Demographic details for the participants is presented in Table 1.

**Table 1:** Characteristics of women participating in stage one (*n* = 10)

		Range	Mean
Age (years)		25–43	33
Gestation (weeks)		31–39	35
		N	
Marital status	Single	2	
	Co-habiting	3	
	Married	5	
Previous children	Primiparous	3	
	Multiparous^a^	7	

Note. ^a^1 previous child. Modal gestation 37 weeks

#### Stage two

All midwives (*n* = 13) had worked within a consultant midwife role for at least a year (range 1–18 years) and regularly supported women with a fear of childbirth. Their more general experience of midwifery ranged up to 30 years.

Recruitment continued until the data reached saturation: for women (*n* = 10) and for consultant midwives (n = 13).

### Materials (interview guides)

For the interviews with women, open questions enquired generally about women’s fears for childbirth, the main elements of their fear and what impact their fears had on their feelings about their pregnancy and their daily life. Participants were also asked about the type of questioning that would enable them to disclose a fear of childbirth during antenatal care and what barriers they might perceive when trying to share their fears. The interviews with midwives focused on professional perspectives on the key elements of the fears that women report and the impacts of these for women. Interviews also incorporated additional questions on optimal methods for encouraging women to disclose their fears, timing of screening women in antenatal care and perceived barriers to implementing a screening tool. Interviews with both groups lasted up to 60 min. The interview topic guides for women and midwives are provided as Additional files 1 and 2 respectively.

### Procedure

#### Stage one

Potential participants were identified as eligible and given information about the study from the consultant midwife and asked whether they would like to receive further information from the researcher. Details of the study were also presented on the LWHFT website and LWHFT social media websites (Twitter, Facebook) to ensure that all pregnant women were given the opportunity to read about the study and were able to contact the researcher directly to enquire about participation. The researcher then contacted women to provide further information about the study. On receipt of consent, the researcher arranged a suitable time to conduct the interview.

#### Stage two

Recruitment took place via snowball sampling and the UK Consultant Midwife network. Midwives interested in hearing more about participating in an interview were asked to contact the researcher for further discussion.

### Analysis

Interviews were audio recorded and transcribed ad verbatim. Each interview was analysed using thematic analysis [25] to identify key concepts associated with fear of childbirth. The most simplistic form of thematic analysis was used as we solely wished to identify the range of FOC. Each transcript was re-read and coded line by line to identify and extract the descriptive information. These descriptions were tentatively grouped into initial themes by KB which were then discussed with the core team who reviewed the labels and evidence throughout the process relabelling and refining final themes as appropriate. Analysis and synthesis across individuals was completed by KB sequentially for primiparous women followed by multiparous women and subsequently consultant midwives. To increase rigor, two interviews were double coded (KB & PS). All iterations of themes were verified by all 4 authors. The researcher (KB) was blind to the meta-synthesis utilised in the final integration as outlined below prior to the completion of interview analyses.

The themes derived from Stages One and Two were then reviewed by the research team (KB KS GH and PS) alongside those generated from a recently conducted meta-synthesis [26], which examined the content and moderators of women’s fears for giving birth as reported by women in the wider literature. Reviewing the themes generated from the present research with those identified as reported by women in the wider qualitative literature enabled the integration of findings representing key elements of fear of childbirth.

In order to identify key elements of the construct, an a priori decision was implemented whereby final themes included only those elements supported by 2 of the 3 sources (women’s interviews, consultant midwife interviews or meta-synthesis). Again, to ensure priorisation of women’s voices, at least one of the sources had to be grounded in women’s accounts of their fear (the women’s interviews or the meta-synthesis, which had only included papers derived from primary evidence from women themselves).

## Results

### Step one: themes generated from women

#### Consideration of findings from primiparous (P) and women and multiparous (M) women

As there was a possibility that fears might differ between primiparous and multiparous women these were initially analysed separately. Interviews with primiparous (P) women (*n* = 3) generated 8 initial themes (areas of fear). Our purpose was to identify all potential areas at this point. Of these, all 3 women shared 6 intial themes of fear of childbirth: 1. Fear of inability to cope with the pain, 2. Fear of my body’s inability to give birth, 3. Fear of harm or stress to the baby, 4. Fear of the unpredictability of childbirth, 5. Fear of my lack of ability to plan and 6. Fear of harm to self. Two of the primiparous women also reported 7. Fear of long-term implications of damage from labour and childbirth. One woman was fearful of 8. Not being ‘heard’ during labour or having an ability to influence what happens.

All 8 initial themes generated from primiparous women were reflected in the analysis of information from multiparous (M) women with the addition of 3 further initial themes; 9. Fear of being abandoned/alone in labour and childbirth, 10. Fear of length of labour and 11. Fear of intervention (including any processes that made them feel ‘done to’). Through discussion with the full team it was clear that ‘Fear of length of labour’ reflected the same concerns as ‘Fear of inability to cope with the pain’, as often the focus was fear of a long or a short labour that is either associated with long and laborious labouring process or a fast and acutely painful labour. Therefore, it was agreed that only ‘Fear of being abandoned/alone in labour and childbirth’ and ‘Fear of intervention (including any processes that made them feel ‘done to’)’ were additional initial themes.

As all initial themes identified by primiparous women were subsequently confirmed by multiparous women the team agreed it was appropriate to fully integrate the analysis whilst ensuring all key elements from both groups continued to be represented.

**Fig. 1:**
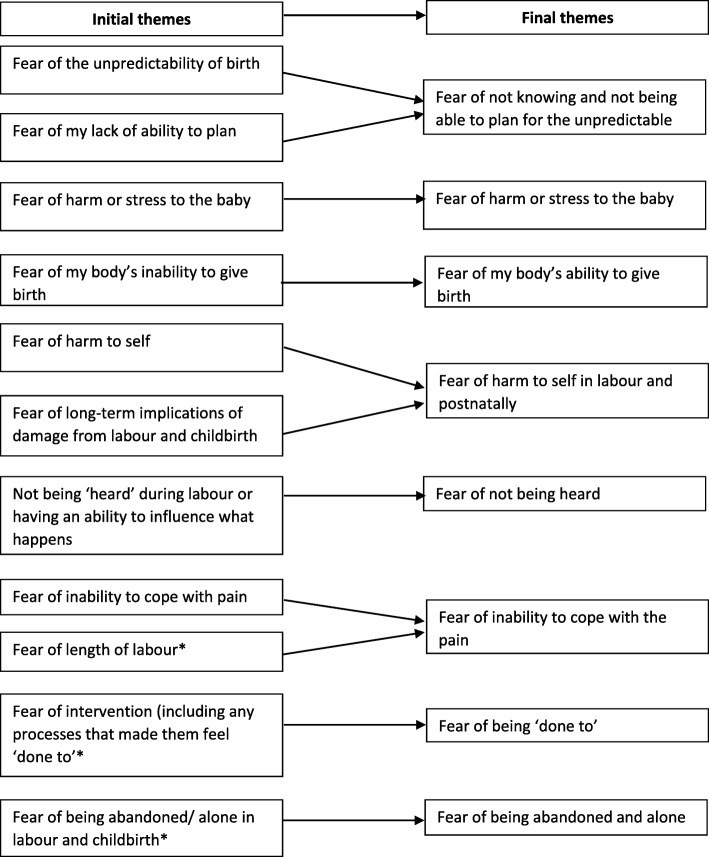
Representation of thematic process for multiparous and parous women. Note. *fears solely identified by multiparous women. All other fears identified by multiparous and primiparous women

All data was therefore reanalysed using the full data set and a set of eight final themes from all the women’s interviews were identified. Whilst these clearly reflect much of the original analysis points of conceptual overlap enabled a reduction in number from the original 11 initial themes to 8 final themes. Final themes and their derivation were as follows: 1. Fear of not knowing and not being able to plan for the unpredictable (from related to previous categories of Fear of the unpredictability of childbirth and Fear of my lack of ability to plan), 2. Fear of harm or stress to the baby 3. Fear of inability to cope with the pain 4. Fear of my body’s ability to give birth (related to previous category Fear of my body’s inability to give birth) 5. Fear of harm to self in labour and postnatally (related to previous categories Fear of harm to self and Fear of long-term implications of damage from labour and childbirth) 6. Fear of being ‘done to’ (related to previous category Fear of intervention including any processes that made them feel ‘done to’) 7. Fear of not being heard (related to Not being ‘heard’ during labour or having an ability to influence what happens) and 8. Fear of being abandoned and alone (related to previous category Fear of being abandoned/alone in labour and childbirth). The process of theme generation from initial themes to final themes is presented for women in Fig. 1.

### Step two: themes generated from midwives (C)

The first iteration of analysis from the consultant midwives (C) interviews generated 13 initial themes: 1. Control (which incorporated a lack of control over the situation and a fear of an internal loss of control), 2. Fear of not receiving the care they would like (which incorporated not being listened to, not being involved in decision making and a general lack of trust in healthcare providers), 3. Fear of the unknown/unpredictability, 4. Fear of pain, 5. Fear of the length of labour (which included both long and quick labours), 6. Fear for the safety of the baby (from harm of the baby through to death of the baby), 7. Indeterminate fear (which included not being sure of what they were afraid of and not establishing the cause of the fear), 8. Fear of the same thing happening again, 9. Fear of induction, 10. Fear of intervention, 11.Physical damage from the birth (including damage to the vagina, damage to the perineum, tearing or stitches), 12. Fear of death and 13. Fear of things going wrong.

**Fig. 2:**
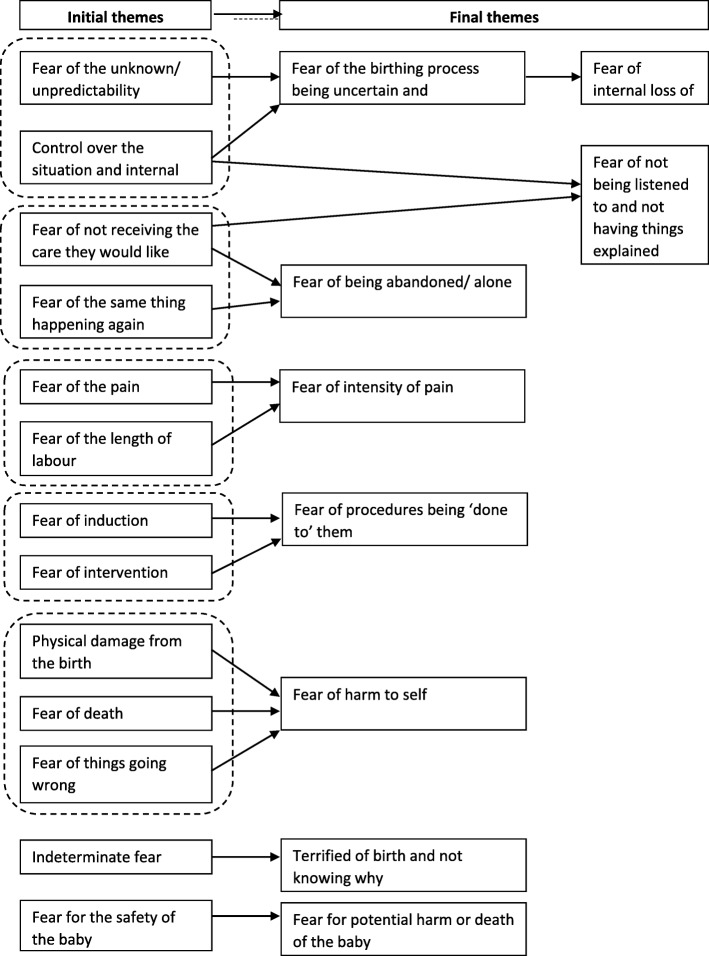
Representation of thematic process for consultant midwives. Note. Initial groupings of categories are indicated via internal dashed boxes

Through team discussion and further iterations of the data these initial themes were streamlined into 9 final themes: 1. Fear of the birthing process being uncertain and unpredictable, (which amalgamated initial themes 1. Fear of a lack of control over the situation and initial theme 3. Fear of the unknown/unpredictablity, 2. Fear for potential harm or death of the baby (which reflected initial theme 6. Fear for the safety of the baby in the first iteration), 3, Fear of intensity of pain (which incorporated both initial theme4 Fear of inability to cope with the pain and initial theme 5 Length of labour as one main theme, as the themes were conceptually linked) 4. Fear of harm to self (this theme expanded to incorporate initial theme 13 when things going wrong in labour, and initial themes 11 and 12 with the woman being hurt, injured or dying, as well as post birth damage), 5. Fear of procedures being ‘done to’ them (this theme amalgamated initial theme 9 Fear of induction and initial theme 10 fear of intervention in general), 6. Fear of not being listened to and not having things explained (this theme was streamlined from initial theme not receiving the care they would like and a fear of their loss of influence over decision making processes and choices), 7, Fear of being abandoned/alone (This theme was created from the previous initial theme 8. Of the same thing happening again, as many women had felt abandoned and alone during their previous labour and birth, and initial theme 2 not receiving the care that they would like), 8. Fear of internal loss of control (this theme was generated from initial theme1 Control but was streamlined to focus more on the internal loss of control rather than loss of control of the situation), 9. Terrified of birth and not knowing why (this theme was initial theme 7 in the first iteration and remained the same). The process of theme generation from initial themes to final themes is presented in Fig. 2.

### Key elements for fear of childbirth

To ensure appropriate content validity, each theme had to reflect women’s own voices and be confirmed by one of the other sources. Therefore, as women’s voices were represented in both the interviews with women and the meta-synthesis [1], each key element is supported by one of these sources plus an additional source of evidence.

Therefore, overall, 10 themes which could be considered key elements for fear of childbirth were identified (Table 2). Seven themes were identified from the women and supported by the consultant midwives: one was generated from the women and supported by the women’s reports in the meta-synthesis and two additional themes were generated by the meta-synthesis and were supported by the consultant midwives. This derivation is shown in Table 3 which shows all themes from all three sources. Each key element is in greater detail below.

**Table 2:** Key elements of women’s fear of childbirth

	Element	Source
1	Fear of not knowing and not being able to plan for the unpredictable	W, CM, MS
2	Fear of not having a voice in decision making	W, CM, MS
3	Fear of harm or stress to the baby	W, CM, MS
4	Fear of inability to cope with pain	W, CM, MS
5	Body’s ability to give birth	W, MS
6	Fear of harm to self in labour and postnatally	W, CM, MS
7	Fear of being ‘done to’	W, CM
8	Fear of being abandoned/alone	W, CM, MS
9	Fear of internal loss of control	CM, MS
10	Terrified of birth and not knowing why	CM, MS

Note. W: interviews with women; CM: interviews with consultant midwives; MS: meta-synthesis

**Table 3:** Themes generated by women, consultant midwives and the metasynthesis

Final 10 elements	Themes generated by women in the study	Themes generated by consultant midwives	Themes generated by the Metasynthesis
1. Fear of not knowing and not being able to plan for the unpredictable	Fear of not knowing and not being able to plan for the unpredictable	Fear of the birthing process being uncertain and unpredictable	The unpredictability of childbirth
2. Fear of harm or stress to the baby	Fear of harm or stress to the baby	Fear for potential harm or death of the baby	Potential for injury or harm *Health and safety of the baby*
3. Fear of inability to cope with pain	Fear of inability to cope with pain	Fear of intensity of pain	Fear of pain
4. Fear of harm to self in labour and postnatally	Fear of harm to self in labour and post-natally	Fear of harm to self	Potential for injury or harm *Personal health and safety*
5. Fear of being ‘done to’	Fear of being ‘done to’	Fear of procedures being done to them	Potential for injury or harm *Complications and interventions*
6. Fear of not having a voice in decision making	Fear of not being heard	Fear of not being listened to and not having things explained *Loss of influence over decision making and choices*	Interactions with care providers
7. Fear of being abandoned/alone	Fear of being abandoned/alone	Fear of being abandoned/alone	Interactions with care providers
8. Body’s ability to give birth	Body’s ability to give birth		Fear of capacity to give birth *Physical capacity to give birth*
9. Fear of internal loss of control		Control *Fear of internal loss of control*	Losing control
10. Terrified of birth and not knowing why		Terrified of birth and not knowing why	Generic fear of unknown

## Fear of not knowing and not being able to plan for the unpredictable

Women reported that they were fearful of the unpredictable nature of birth and were concerned that they are unable to plan for this unpredictable event. For primiparous women, this presented in the form of a lack of knowledge or experience of how to manage the event, for multiparous women it was more an understanding of how unpredictable the birthing process can be, given their previous experience. This was supported by the consultant midwives under the theme of ‘Fear of the birthing process being uncertain and unpredictable’. Many of the midwives speculated about whether the fear is generated because women control so many aspects of their day-to-day life but cannot control the birthing process.

For some, the opportunity to create a detailed birth plan abated this fear. However, for many, the unpredictability of who would action the birth plan or be on duty that day didn’t reduce (and in some cases increased) this sense of unpredictability.

*‘I am still scared and I still don’t like the idea of unpredictability in anything.*

*Predictability brings control.’ (P19).*

*‘I don’t know how the births going to go or what’s going to happen again, you know you’re not in control of it, it’s just dead scary.’ (M21).*

*‘My view is that women have so much control over every aspect of their life, they find it – childbirth a really hard thing to do. In terms of them not being able to predict what’s going to happen, not, not feeling as if they’ve got control. It, It, it, it’s hard to package into a life where you manage everything else so tightly’. (C3).*

## Fear of not having a voice in decision making

Women were fearful of not being involved in decision making throughout the labour and birthing process. This was mainly identified by multiparous women, however one primiparous woman also identified this as a fear. Despite being able to write a detailed birth plan and discuss their hopes and fears about their birth with the professionals beforehand, there was a fear that during the birthing process their requests would be missed. This theme was identified by the consultant midwives as ‘Fear of not being listened to and not having things explained - *Loss of influence over decision making and choices’* and they spoke of women (particularly multiparous women) reporting that they were not involved during their previous labour when decisions were being made.

‘But when I spoke to the midwife and told her what *** (consultant midwife) had said, she said ‘oh I think you should make sure that that is in your birth plan and you keep saying it and keep saying it and if they don’t listen that you do speak to somebody higher’. So even though *** (consultant midwife) had reassured me then I felt a bit… scared again.’ (M8).

*‘So the example that I have had really are women who have wanted to remain upright and mobilising but they often come to me and say ‘I wasn’t able to move around, I wasn’t given a choice… I wanted to do X, Y and Z and you wouldn’t let me’ (C18).*

## Fear of harm or stress to the baby

Women reported a fear of the baby being hurt in the process of labour and childbirth. For them, harm to the baby ranged from the baby being distressed during the labour and delivery to the baby being physically harmed in the process. This was also identified by the consultant midwives ‘Fear for potential harm or death of the baby’. The consultant midwives tended to report a greater fear of the physical harm to the baby rather than distress to the baby and also reported a fear of the baby dying, which was not identified by the women in this study.

*‘I am scared for the baby. I don’t want the baby to go through anything – from like getting pulled out by the forceps or the suction- the cup- or anything, I’d rather…try and…avoid whatever I can for them.’ (P1).*

*‘So I think it is the fear of losing the baby as well when in labour or having a damaged baby’. (C10).*

## Fear of inability to cope with pain

Fear of pain not only included women’s ability to manage the intensity of the pain but also their concern about whether they would receive the appropriate level of pain relief for them to cope. Fear of pain was also identified by the consultant midwives who noted that multiparous women often remember the pain from last time, but it is variable as to whether that then becomes the main focus of their fear in the current pregnancy. They also reported that for some women their fear of pain is not specific to childbirth; more a broad fear of being unable to cope with pain in general.

*‘I am fearful – I am really scared of the pain’ (P3).*

*‘And a lot of it is around the pain relief as well, you shouldn’t be expected to be in that amount of pain ever.’ (M20).*

*‘I mean you do get the women that come in and go ‘I can’t, I can’t cope with pain, I’m an absolute wuss, I just want to have an epidural and that’s fine’ (C11).*

## Body’s ability to give birth

This element was identified by the women and within the meta-synthesis but not by consultant midwives. The women reported a fear of their body’s physical capacity to birth the baby. Their fears included their own body size, their baby’s size/positioning and their physical strength.

*“I feel that my pelvis is really small and I’ve got a narrow pelvis.” (P 3).*

*“I’m terrified in case the head just – if anything goes wrong – the head was to turn or I couldn’t get her out.” (M6).*

## Fear of harm to self in labour and postnatally

This element of fear captures the belief that something ‘bad’ will happen in labour. The term ‘harm’ encapsulated a range of fears, from feeling generally unsafe during labour through to a fear of dying in labour and childbirth. This was reflected in the consultant midwives interviews too under the theme of ‘Fear of harm to self’, and this theme often referred to women being fearful of being physically damaged during birth or dying.

*“And I think about the day that I have to go in for the section and I look at my little boy and I’m thinking am I really not going to see him again, am I really not going to see him again? (sobbing).” (M22).*

*“They just have been getting this drip, drip, drip effect of negativity and if the only thing is negative, that birth is this horrible experience that is going to traumatise me and wreck my body and I could die…” (C7).*

The fear of harm to self, included a sub-theme of potential post-natal complications. Women and consultant midwives both reported that women fear that the labour and birth might lead to a slow and painful healing process, one that creates irrevocable damage to the woman’s body.

*“My relationship with my husband is important to me and I worried that it would change after birth” (P19).*

*“And like the last thing I want is when I come home, I can’t pick my little girl up and I can’t give her all the big cuddles and everything because I have got this big scar on my stomach.” (M21).*

*“They’re worried that their perineum and their ongoing sexual health will be damaged” (C3).*

## Fear of being ‘done to’

This refers to a more general fear of intervention of any sort, ranging from vaginal examinations to instrumental deliveries. Women referred to feeling like ‘a piece of meat’. This theme is characterised by the words ‘done to’ as this was often the phrase women used about these interventions, as if they were not a participant in the process. This theme reflects more than just a lack of communication, but a feeling of violation of their body during procedures. This was also reflected in consultant midwives reports of ‘Fear of procedures being done to them’.

*“I am absolutely petrified of needles. Petrified. So I was like, do not let me get that epidural whatever I say no matter how desperate I am don’t let me get it.” (M13).*

*‘So, having all of that intervention potentially, lots of examinations, lots of monitoring, drips, you know, not getting any sleep. When you end up going down that sort of pathway of interventions, people end up not being able to eat, not being as mobile and it is just a horrible process.’ (C16).*

## Fear of being abandoned/alone

This element, for the women, was mainly around being physically left alone by staff when they did not wish to be and also a fear that their chosen birth partner wouldn’t be able to be by their side when they needed them. This was also identified by the consultant midwives as ‘fear of being abandoned/alone’ but was more frequently referring to staff members (rather than birth partners) when they felt they needed support. Within the meta-synthesis this element also included an aspect of being psychologically alone during labour and birth.

*“(if has a caesarean-section) So at least I know I won’t be left in a room just dealing* with it” (M8).

“*** (husband) works in (city). So and I know that obviously with winter coming and the traffic is all bad, the M62 is horrendous and with my mum dying I’ve got no mum” (M20).

*“…‘I was left you know, just left with this baby and the midwife left the room’ and so we are trying to you know, it is about getting the midwife to stay in the room and do the computers and the notes in the room with the women, so the women have people there for support” (C11).*

## Fear of internal loss of control

Although this was not identified by the women in this study, it was identified by the consultant midwives and also within the meta-synthesis. It refers to a feeling about an internal turbulence and loss of self-control, where the woman no longer feels she is able to manage herself, which can lead to her battling internally throughout the labour and birth.

*“They just don’t like the thought of being out of control and knowing that they are out of control.” (C5).*

## Terrified of birth and not knowing why

Although this was not captured in interviews with the women in this study, this was reported by the consultant midwives and was also noted in the meta-synthesis. It is an element which represents a more general feeling of fear without any understanding of the specific reasons for feeling fearful, and a lack of root cause for the fear of labour and childbirth.

*“There’s very rarely a root cause that I find, even though I spend as much time as the woman wants talking to them they are often unable to articulate why this fear is - it’s just there” (C3).*

*“But some people are genuinely frightened and there are lots of women that are frightened but for lots of… you know, the unknown reasons” (C1).*

### Women’s views about when FOC should be assessed

During the women’s interviews, the women were also asked their views about when the appropriate time to complete an assessment for FOC would be or when any discussions around FOC should take place and the overwhelming majority of women felt that questions should be asked about fear as early as possible to ensure appropriate support can be put in place:

*“And I think we should be asking women right at the beginning about their fears because I think when you find out you are pregnant it is the thing you think about the most. Because obviously I knew I was pregnant as soon as it had happened, so it’s kind of you start thinking about it, you start thinking again what happened last time…so you do think about it straight away yeah. Definitely need to talk to women about this” (M20).*

*“Something around someone saying ‘well have you got any concerns about delivery’ at a very, very early stage might help. Because I think I was kind of, I think that at about 6 weeks I was in tears with the community midwives talking about delivery. So I was, right from the get go, I was thinking – and maybe I am unusual in that respect, I don’t know. I don’t know what other ladies have said but I kind of think that even if – even if that was identified quite early on and you’ve got kind of those conversations with the consultant midwife much earlier I think that might help”. (M5).*

*“I think as early on as you can ask them open questions to understand if there is any fears that you can deal with and book people in with the appropriate people and have time to do that then. For me that’s the best.” (M22).*

*“Not being able to discuss until you are 30 whatever weeks – it’s too late.” (M13).*

### Midwives views about when FOC should be assessed

During the consultant midwife interviews, midwives were asked their views about when the appropriate time to assess for FOC would be. All midwives felt it should be completed before 20 weeks and the majority felt it should be as early as possible (generally around booking time):

*“So for me the sooner we can support women, see women to talk about it, the better it is because we can start to manage those expectations. We can manage it in sizeable chunks that are led by the woman not me.” (C9).*

*“Send that woman to see someone early in the pregnancy because the longer you dismiss it and ignore it the greater the worry and anxiety for that woman and the shorter the amount of time.” (C15).*

For those who suggested later than booking (i.e. 16 weeks or 20 weeks), it was often due to practical issues in terms of the amount of paperwork that already needs to be completed at booking. Those midwives suggested that a further demand on this already busy appointment might be impractical.

*“Perhaps at 16 weeks. There is so much going on at booking, there is and you know you can acknowledge fear and say ‘this is something you know we will explore in more detail at your next appointment’ perhaps say ‘have a think, here’s the list of questions’ you know, do some sort of preparation for that conversation, so you’re acknowledging that this women is frightened of birth in some way. Yeah but I think perhaps 16 weeks is a good time to start.” (C16).*

## Discussion

There is an emerging emphasis on the need for a clear, usable definition for the fear of childbirth construct [2, 17] which has established validity. Findings from this qualitative investigation integrates accounts of women experiencing high levels of fear about giving birth, perspectives of consultant midwives experienced in supporting women experiencing fear of childbirth, and women’s accounts of their fear as reported in the wider qualitative literature (from a recently completed meta-synthesis) [26] to establish ten key elements underpinning and present within the fear of childbirth construct.

Parallels between the perspectives of both primiparous and multiparous women, consultant midwives, and findings reported in the wider qualitative literature emphasise the centrality of the key elements in the fear of childbirth construct. Fear of the unknown highlights the role of uncertainty and unpredictability in the birth process eliciting fear. Not feeling heard during the birthing process, fearing harm or stress to the baby, concerns over coping with pain, feeling ‘done to’ or abandoned during the birth were elements strongly endorsed by women and midwives as important elements of fear of childbirth. Several of these elements have also been identified as important for the identification of fear of childbirth following a recent review of quantitative literature [23].

There were however differences identified between each of the sources used to develop the construct. It is interesting to note that fear of their body’s capacity to give birth was only reported in women’s interviews and the meta-synthesis^26^, and not in the midwives’ interviews; indicating the importance of this element within the definition for FOC and any screening tool, as it currently unlikely to be a question that is specifically asked by midwives.

The element of ‘fear of internal loss of control’ was only captured by the accounts of women reported in wider qualitative studies via the meta-synthesis and thus warrants further exploration. Within the meta-synthesis, women reported concerns over losing physical or emotional control during the birth, and this leading to either not ‘performing well’ or not co-operating with staff [26]. However this was not an element of fear reported by women interviewed as part of the present study, indicating that this aspect of women’s fears requires further exploration.

The element of generic fear of the unknown was an interesting theme and raised some questions within the core team during the analysis process. Given that anxiety and fear is often coupled with a significant amount of avoidance [27], this might be more reflective of the challenges of truly engaging these women in exploring their fears rather than an element of fear itself; however, this requires further exploration. For example, previous qualitative investigations have identified that women experiencing high levels of FOC may attempt to avoid thoughts or talking about their fears [28, 29], and it is plausible to suggest that this may have resulted in the generic element for fear of the unknown. Findings also highlight perspectives of women and midwives regarding the optimal timing for asking about fear during pregnancy with both women and midwives report that early identification is preferable. Supporting women to discuss fears as early as possible during pregnancy will enable timely access to support to help mitigate or alleviate concerns, and potentially prevent women experiencing high levels of fear of childbirth throughout their pregnancy. Further research is required to identify feasible and acceptable methods of introducing strategies to identify FOC at an early stage of antenatal care.

### Strengths and limitations

A particular strength of this study is that it reflects the voices of both primiparous and multiparous women and each element remains as close to the women’s language and phraseology as possible (e.g. ‘done to’). This will enable women who are fearful to identify with these key elements in future work and find them truly reflective of their feelings about labour and childbirth. There was a high degree of consensus between the women and the consultant midwives’ elements for fear of childbirth, emphasising the utility of these elements for inclusion in a future screening tool. It is also interesting to note the homogeneity in fears reported by primiparous and multiparous women, however it must be noted that the current sample included only three primiparous women. The sample is self-selecting and given the nature of the interviews it is highly likely that the interviews did not capture the views of those women who are pregnant and too afraid to talk about their feelings. Nor does it capture those who are avoiding becoming pregnant because of their fear of childbirth.

All women were in their 3rd trimester when they were interviewed, given that women’s fear of childbirth increase in the 3rd trimester [30], future studies might consider reviewing women’s fear of childbirth in all 3 trimesters. Also, this study only included 3 primiparous women, and although the themes generated were reflected in both groups, studies have suggested that the content of fears of childbirth might be different for primiparous and multiparous women [31], therefore this might warrant further exploration in future studies. However, themes were reviewed in parallel to those obtained from a meta-synthesis of women’s accounts of their fears for giving birth, where the views of both multiparous and primiparous women were included [26].

The role of partner relationship may be of relevance in relation to fears of childbirth too [32], and the marital status of the participants in this study were noted and reflected a typical pattern in pregnant women with the majority being married or cohabiting.

### Relevance to clinical practice

Clear identification of women who are fearful of childbirth will allow healthcare professionals to activate an early and effective pathway of care for these women. However this requires appropriate measurement tools that must be derived from a clearly articulated and evidence based construct for fear of childbirth [17]. The current study combines information from three sources to identify key elements of central to a construct of fear of childbirth as reported by women themselves. By defining the key elements of fear of childbirth, the development of relevant and culturally appropriate measurement tools with high content validity, or examination of existing tools, can be facilitated. These key elements also provide an insight into what should be included in packages of care to ensure effective and relevant support for these women. The next stage of the work is to assess women’s understanding of items in existing tools and to map the emergent elements from this study across existing ways of assessing FOC.

Although development of a culturally appropriate definition of FOC for UK is necessary, there remains a need to develop current understanding regarding the definition, identification and support for women experiencing FOC on an international basis. Whilst there will be differences between populations regarding the focus and content of fears, it is plausible to suggest that there will also exist parallels; as evidenced by those elements identified in Sheen and Slade’s^26^ meta-synthesis where reports of fears on an international basis were integrated. Findings from the present study may therefore contribute to understanding the key elements of FOC experienced by women regardless of cultural context.

## Conclusions

This study identified ten key elements present in women’s accounts of their fear of childbirth, supported by the accounts of midwives with experience of providing support in this context. There was a clear preference to implement methods of identifying fear of childbirth early in pregnancy. These findings can be used to inform development of comprehensive and culturally appropriate methods of identifying fear of childbirth during pregnancy, and also hold implications for the shaping of supportive interventions aimed at reducing women’s distress and fear of childbirth prior to birth.

## Additional files

Additional file 1: Topic guide for fear of childbirth semi-structured interview with women (DOCX 29 kb)

Additional file 2: Topic guide for fear of childbirth semi-structured interview with consultant midwives (DOCX 18 kb)

## Abbreviations

FOC: Fear of childbirth
LWHFT: Liverpool women’s hospital NHS foundation trust
