# Supplementary material for: Establishing a valid construct of fear of childbirth: findings from in-depth interviews with women and midwives
Source: BMC Pregnancy Childbirth. 2019 Mar 18;19:96. doi: 10.1186/s12884-019-2241-7 (PMC6423809; doi:10.1186/s12884-019-2241-7)
Supplement: Supplementary file 1 — Topic guide for fear of childbirth semi-structured interview with women (DOCX 29 kb) [file 12884_2019_2241_MOESM1_ESM.docx]

Additional File 1. Topic guide for fear of childbirth semi-structured interview with women

The interview will be semi structured, guided by the questions detailed below.

1. Can you tell me about the worries, concerns or fears that you have about childbirth?
   1. *What would make your childbirth the best it could be?*
   2. *What would make it the most difficult it could be?*
2. Tell me a bit about when these fears first began
   1. *How have they changed/ remained the same over time?*
3. In what ways do you think these feelings affect you?
   1. *In daily life*
   2. *Relationships*
   3. *How you feel about yourself*
   4. *How you feel about your pregnancy*
4. What sort of things have made things easier for you?
5. What sort of things have got in the way?
6. How could care in pregnancy be provided *to improve things for you?*
   1. *How do you think that midwives can be the best help to you*?
   2. *How easy/difficult has it been to talk about your concerns with staff*
7. Is there anything else important relating to what we have spoken about today that you would like to comment on?
