# Supplementary material for: Establishing a valid construct of fear of childbirth: findings from in-depth interviews with women and midwives
Source: BMC Pregnancy Childbirth. 2019 Mar 18;19:96. doi: 10.1186/s12884-019-2241-7 (PMC6423809; doi:10.1186/s12884-019-2241-7)
Supplement: Supplementary file 2 — Topic guide for fear of childbirth semi-structured interview with consultant midwives (DOCX 18 kb) [file 12884_2019_2241_MOESM2_ESM.docx]

Additional File 2. Topic guide for fear of childbirth semi-structured interview with consultant midwives

The interview will be semi structured, guided by the questions detailed below.

1. Can you tell me about your experiences of providing support to women who are fearful of birth?
2. *How many women do you provide support for?*
3. *How much need do you think there is for support to be provided?*

1. What sort of things surrounding birth do women report as fearful to you?
2. *What sort of things do you think make women more fearful?*

1. What do you think are the key elements of women’s fears for giving birth?

1. How easy do you find it to support women who are fearful of birth?
2. *What sort of things get in the way?*

1. How do you think that care for women who are fearful of birth could be improved*?*
2. *What sort of questions do you think could be asked?*
3. *When do you think the best time to approach women about this would be?*
4. *Is there anything else you feel that could be done to improve the support available for women?*

1. What challenges do you think there are when providing support for women who are fearful of birth?

1. Is there anything else important relating to what we have spoken about today that you would like to comment on?
